# Free-running cardiac and respiratory motion-resolved 5D whole-heart coronary cardiovascular magnetic resonance angiography in pediatric cardiac patients using ferumoxytol

**DOI:** 10.1186/s12968-022-00871-3

**Published:** 2022-06-27

**Authors:** Christopher W. Roy, Lorenzo Di Sopra, Kevin K. Whitehead, Davide Piccini, Jérôme Yerly, John Heerfordt, Reena M. Ghosh, Mark A. Fogel, Matthias Stuber

**Affiliations:** 1 Department of Diagnostic and Interventional Radiology, Lausanne University Hospital (CHUV) and University of Lausanne (UNIL), Rue de Bugnon 46, BH-8-84, 1011 Lausanne, Switzerland; 2 Division of Cardiology, Department of Pediatrics, The Children’s Hospital of Philadelphia, University of Pennsylvania Perelman School of Medicine, Philadelphia, USA; 3 Advanced Clinical Imaging Technology, Siemens Healthcare AG, Lausanne, Switzerland; 4 Center for Biomedical Imaging (CIBM), Lausanne, Switzerland

**Keywords:** 5D, Free-running, Coronary MRA, Pediatric patients, Congenital heart disease, Free-breathing, Self-gating, Whole-heart, Ferumoxytol

## Abstract

**Background:**

Coronary cardiovascular magnetic resonance angiography (CCMRA) of congenital heart disease (CHD) in pediatric patients requires accurate planning, adequate sequence parameter adjustments, lengthy scanning sessions, and significant involvement from highly trained personnel. Anesthesia and intubation are commonplace to minimize movements and control respiration in younger subjects. To address the above concerns and provide a single-click imaging solution, we applied our free-running framework for fully self-gated (SG) free-breathing 5D whole-heart CCMRA to CHD patients after ferumoxytol injection. We tested the hypothesis that spatial and motion resolution suffice to visualize coronary artery ostia in a cohort of CHD subjects, both for intubated and free-breathing acquisitions.

**Methods:**

In 18 pediatric CHD patients, non-electrocardiogram (ECG) triggered 5D free-running gradient echo CCMRA with whole-heart 1 mm^3^ isotropic spatial resolution was performed in seven minutes on a 1.5T CMR scanner. Eleven patients were anesthetized and intubated, while seven were breathing freely without anesthesia. All patients were slowly injected with ferumoxytol (4 mg/kg) over 15 minutes. Cardiac and respiratory motion-resolved 5D images were reconstructed with a fully SG approach. To evaluate the performance of motion resolution, visibility of coronary artery origins was assessed. Intubated and free-breathing patient sub-groups were compared for image quality using coronary artery length and conspicuity as well as lung-liver interface sharpness.

**Results:**

Data collection using the free-running framework was successful in all patients in less than 8 min; scan planning was very simple without the need for parameter adjustments, while no ECG lead placement and triggering was required. From the resulting SG 5D motion-resolved reconstructed images, coronary artery origins could be retrospectively extracted in 90% of the cases. These general findings applied to both intubated and free-breathing pediatric patients (no difference in terms of lung-liver interface sharpness), while image quality and coronary conspicuity between both cohorts was very similar.

**Conclusions:**

A simple-to-use push-button framework for 5D whole-heart CCMRA was successfully employed in pediatric CHD patients with ferumoxytol injection. This approach, working without any external gating and for a wide range of heart rates and body sizes provided excellent definition of cardiac anatomy for both intubated and free-breathing patients.

**Supplementary Information:**

The online version contains supplementary material available at 10.1186/s12968-022-00871-3.

## Background

Coronary cardiovascular magnetic resonance angiography (CCMRA) is employed in pediatric patients with congenital heart disease (CHD), where the accurate assessment of anatomic structures and coronary vessels constitutes a fundamental requirement for interventional planning. Conventional clinical practice includes first-pass magnetic resonance angiography and 2D cardiac cine acquisitions, which typically require lengthy scanning sessions with repeated breath-holding, under anesthesia and respiratory intubation at times [1]. A specialized team of technologists and physicians is oftentimes mandatory for accurate scan planning in the presence of relatively complex anatomy. It has also been reported that such methods may fall short of providing sufficient spatial and temporal definition for structures such as chordae or subtleties of valve morphology [1, 2]. As a result of these challenges, several recent studies have proposed methods for whole-heart 3D pediatric cardiac CCMRA with submillimeter isotropic spatial resolution and motion resolution along the cardiac cycle in an effort to provide a comprehensive and easy-to-use acquisition. One example of this powerful approach was introduced by Han et al. with the four-dimensional (4D) multiphase steady-state imaging with contrast enhancement (MUSIC) technique [3]. This electrocardiogram (ECG)-triggered spoiled gradient recalled echo (GRE) sequence at 3T provided five to eight 3D cardiac phase images and respiratory gating was ensured through the air pressure signal of the mechanical ventilator. Further developments of this framework included: a rotating Cartesian k-space (ROCK) trajectory enabling retrospective data binning; the use of cardiac and respiratory self-gating (SG) surrogate signals to substitute the ECG and the mechanical ventilator motion signals; a nonlinear iterative reconstruction algorithm combining parallel imaging and compressed sensing (CS) [3–5]. The diagnostic performance and clinical impact of 4D MUSIC has already been extensively evaluated in cohorts of neonates and infants with CHD providing accurate, simple, and safe dynamic 3D imaging of cardiovascular anatomy [6]. However, this approach presents some limitations: (1) respiratory intubation during anesthesia is still needed in these studies; (2) the time efficiency is limited (about 40%), as some data are discarded and do not contribute to the final reconstruction; (3) manual interactions for scan parameter adjustment are sometimes needed to adapt to the subject-dependent respiratory patterns; (4) and the temporal resolution remains to be further improved to account for higher heart rates particularly in pediatric cohorts with 9–12 phases per cardiac cycle resolved in current published protocols [7], since acquiring more frames significantly prolongs scan time.

Recently, the free-running framework [8] was developed for fully SG cardiac- and respiratory motion-resolved 5D whole-heart imaging with high isotropic spatial resolution (1.1 mm^2^) and high temporal resolution (15–30 phases per cardiac cycle). The goal of this work was to translate and exploit the efficiency and simplicity of the free-running framework to provide an automated push-button solution for whole-heart CCMRA in pediatric patients that does not require any breath-holding, navigation, external gating, or triggering device. Similar to previous studies including the aforementioned MUSIC technique [3], we added our technique to an existing clinical protocol used for CHD patients injected with ferumoxytol [2, 4, 6, 9, 10]. Ferumoxytol, which remains in the blood stream for an extended period of time relative to gadolinium-based contrast agents [2, 11, 12] provides more time to invest in the image acquisition and therefore supports imaging with high spatial resolution and high temporal resolution, which is a clear advantage for pediatric patients who have small structures and fast heart rates. We assessed the imaging performance of free-running 5D imaging, using the visualization and sharpness of the coronary artery ostia as a metric for both the achievable spatial and temporal resolution. Finally, 5D imaging was performed in both patients with intubation and patients who were breathing freely in an effort to advance the hypothesis that anesthesia and intubation, which is frequently used to minimize bulk motion and control respiration, may be avoided, thus providing a significant potential improvement of ease-of-use in pediatric CCMRA.

## Methods

### Free-running 5D MRI framework for Pediatric Patients

The study was performed in 18 pediatric cardiac patients (8.0 ± 5.6 years, range less than 1–16 years) at the Children’s Hospital of Philadelphia (Philadelphia, Pennsylvania, USA) on a whole-body 1.5T clinical MR scanner (MAGNETOM Avanto^FIT^, Siemens Healthineers, Erlangen, Germany). The study was approved by the Institutional Review Board and written informed consent was obtained from the legal guardians prior to CMR scanning. The proposed free-running sequence, further described below, was added to an existing clinical protocol wherein each patient received a 4 mg/kg dose of ferumoxytol (Feraheme, AMAG Pharmaceuticals, Waltham, Massachusetts, USA; Rienso, Takeda Pharmaceuticals, London, United Kingdom), injected slowly over 15 min, with monitoring performed every 5 min during injection and 30 min afterwards. Eleven patients were under respiratory intubation and general anesthesia during the exam (*Group 1*, 5.6 ± 4.7 years), while in the remaining 7 patients, data were collected during free-breathing and without anesthesia (*Group 2*, 11.7 ± 5.0 years, p = 0.024). A previously reported prototype free-running (ECG-free, navigator-free) sequence [13] was modified in that the balanced steady state free precession (bSSFP) module was replaced by GRE. Radiofrequency (RF) excitation angles were adapted to the specific application, while all chemically shift-selective fat saturation pulses and ramp-up RF excitations were removed, alleviating specific absorption rate (SAR) burden, and enabling entirely uninterrupted data acquisition. k-Space data were continuously acquired using a 3D golden angle radial sampling pattern following a spiral phyllotaxis trajectory [14]. The continuous acquisition was segmented into multiple interleaves, each one incorporating a readout oriented along the superior-to-inferior (SI) direction to support simultaneous cardiac and respiratory SG [8, 15]. The employed sequence parameters are summarized in Table 1. For each data set, the SG cardiac triggers and the SG respiratory motion signal were extracted from the SI readouts with a previously reported fully automated method [8]. Based on the obtained SG triggers, data were retrospectively sorted into a variable number of cardiac bins (ranging from 13 to 24), depending on the patient specific heart rate, and following previously established temporal resolution guidelines for imaging children with congenital heart disease [16]. The same data were also partitioned into 4 equally populated respiratory motion states according to the amplitude of the SG respiratory signal following previously established methods for respiratory resolved whole-heart imaging [8, 17, 18]. In both cases, the individual readouts were assigned to their respective cardiac and respiratory bins independently from their position in the acquired radial interleave in order to maximize the reconstructed temporal resolution. Finally, 5D (x–y–z-cardiac-respiratory dimensions) motion-resolved images were reconstructed using a previously reported compressed sensing (CS) algorithm that exploits sparsity along both the cardiac and respiratory dimensions [8, 17]. This framework has since been extended, as the CS optimization problem was here solved with an Alternating Direction Method of Multipliers (ADMM) algorithm [19]. In particular, total variation regularization weights along the cardiac and respiratory dimensions were both set to 0.01, the augmented term of the augmented Lagrangian (rho) was fixed to 0.06, and a total of 10 ADMM iterations were performed. All image reconstructions were performed offline in Matlab (MathWorks, Natick, Massachusetts, USA) on a workstation equipped with 2 Intel Xeon CPUs (Intel, Santa Clara, California, USA), 512 GB of RAM, and a NVIDIA Tesla GPU (Nvidia, Santa Clara, California, USA). The reconstruction time for 5D images across all subjects was recorded.

**Table 1:** Sequence parameters

TR	From 3.3 ms to 3.7 ms
TE	From 1.6 ms to 1.7 ms
RF excitation angle	15°
Field of view	From 180^3^ mm^3^ to 220^3^ mm^3^
Samples per readout	192
Spatial resolution (isotropic)	From 0.94^3^ mm^3^ to 1.15^3^ mm^3^
Number of interleaves	5749
Number of readouts per interleaf	22
Total number of readouts	126,478
Total scan time	7.4 ± 0.3 min

To test the imaging framework, small variations to the spatial resolution were chosen prior scanning in consideration of the pediatric patient size resulting in a small variation in total scan time

### Cardiac temporal resolution

Consistent with the previously published free-running 5D imaging approach in healthy adult subjects, data were sorted into cardiac bins of 50 ms duration without view sharing [8, 17]. However, in consideration of the faster heart rate sometimes observed in pediatric subjects and following previously established temporal resolution guidelines for imaging children with CHD [16], for patients with 100 bpm or higher (corresponding to cardiac cycle lengths ≤ 600 ms), a 25 ms temporal resolution was chosen retrospectively during reconstruction to minimize cardiac motion blurring. The capability of retrospectively and flexibly selecting the temporal resolution for image reconstruction is enabled by the free-running framework which uses CS to recover images despite retrospectively changing the undersampling factor for each reconstructed bin. To test whether the temporal resolution could be improved from 50 to 25 ms, one of the datasets was repeatedly reconstructed using a set of different temporal window widths along the cardiac dimension, ranging from 15 to 150 ms (and resulting in an inversely proportional number of bins).

As already reported by previous studies, rapid gradient switching and the magneto hydrodynamic effect may induce unwanted signals overlaid to the ECG recordings and hamper R-wave detection, especially during uninterrupted acquisition protocols [7, 8, 20]. For this reason, the ECG traces recorded during the acquisition were first visually inspected for irregularities. If frequent artifactual signal peaks that were considered inconsistent with the rhythm of the heart were identified, these ECG traces were excluded from further comparison with the SG signals. As an example, and in addition to the regular SG reconstruction, two of the datasets were reconstructed with the artifactual ECG trigger information for comparison. The agreement between the remaining ECG traces and the corresponding SG signals, as well as the percentage of missed ECG & SG triggers, were estimated as described in [8].

### Respiratory motion resolution

To evaluate the respiratory motion resolution in the reconstructed free-running images, the sharpness of the lung-liver interface was measured for all 4 states of the breathing cycle across all subjects. To this end, parametrized sigmoid functions were fitted to the air-tissue interface of the right hemidiaphragm and then normalized to the sigmoid amplitude. In the considered parametrization of the sigmoid, the value of the slope parameter ($\theta$) is representative of the interface’s sharpness in the targeted profile [21]. For each respiratory position in the reconstructed image, the sigmoid function was fitted to 20 manually selected superior-inferior profiles in the coronal plane. Finally, results (median and standard deviation of $\theta$ across all respiratory phases and all subjects) were statistically compared among the two patient sub-groups (intubated vs free-breathing) using a t-test with p < 0.05 considered significant.

### Visibility assessment of coronary arteries

For all 18 subjects, one systolic and one diastolic end-expiratory 3D cine frame were manually selected from the reconstructed 5D datasets that included 52–96 3D images in total depending on the heart rate. In the 36 resulting 3D images (2 frames $\times$ 18 subjects), the visibility or non-visibility of the origin of the right coronary artery (RCA), left main coronary artery (LM), left anterior descending coronary artery (LAD), and left circumflex coronary artery (LCx) was assessed by one of the authors (MS with 30 years of experience in CMR), which represents a relevant metric for the evaluation of CHD in pediatric patients. The same 3D images were then reformatted using Soapbubble to follow the tortuous vessel path of the coronaries [22]. As a surrogate to overall image quality and motion resolution, visible vessel length (*L*) and interface sharpness (*S*) of the RCA, LM + LAD, and LCx were measured on the reformatted images, the coronaries being rather small in diameter and subject to a large amplitude of displacement during the cardiac and respiratory motion. Cases where the coronary origin was not visible were excluded from further analysis. The visible coronaries were then divided into *Group 1* (patients under respiratory intubation) and *Group 2* (free-breathing), and vessel sharpness and length were statistically compared between the two groups using t-tests.

## Results

The acquisition and reconstruction of the 5D datasets succeeded in all 18 subjects and the mean and standard deviation of the reconstruction time for 5D images across all subjects was 7.3 ± 3.1 h with variability attributed to the number of active coil elements and number of reconstructed cardiac bins. Both respiratory and cardiac motion could be resolved with the proposed SG physiologic signal extraction approach and the CS reconstruction framework (Additional file 1). The 5D images obtained from Subject 16 (average cardiac cycle length = 456 ms) that were reconstructed with different, decreasing temporal resolutions can be observed in Additional file 2, where the number of reconstructed bins along the cardiac dimension ranges from 30 to 3. On these images it can be observed that the interface definition of the intracardiac anatomy degrades both at the lowest temporal resolution, as a consequence of the insufficient motion resolution, and at the highest temporal resolution, as a consequence of undersampling artifacts. Among all datasets, 11 were reconstructed with a 50 ms window width along the cardiac dimension (5 intubated, 6 free-breathing—average cardiac cycle length = 794 ± 164 ms), and the remaining 7 with 25 ms (6 intubated, 1 free-breathing—average cardiac cycle length = 516 ± 59 ms, p < 0.001) (Table 2).

**Table 2:** Patient, scanning, and imaging information

Patient	Age (years)	Resp. scanning condition	Heart rate (bpm)/Average RR-interval (ms)	Cardiac temporal resolution (ms)	Deviation between ECG and SG trigger interval duration (ms)
1	4	Intubated	67/894	50	×
2	16	Free-Breathing	81/744	50	8.3
3	9	Intubated	86/605	50	40.5
4	7	Intubated	93/644	50	7.6
5	< 1	Intubated	146/411	25	34.9
6	6	Intubated	108/553	25	10.7
7	12	Free-Breathing	84/715	50	×
8	12	Free-Breathing	107/563	25	×
9	16	Free-Breathing	50/1202	50	65.9
10	15	Intubated	77/777	50	×
11	< 1	Intubated	109/550	25	×
12	9	Free-Breathing	87/693	50	×
13	15	Free-Breathing	67/891	50	×
14	11	Intubated	71/850	50	68.2
15	2	Free-Breathing	96/625	50	×
16	1	Intubated	132/456	25	11.2
17	6	Intubated	115/523	25	29.4
18	3	Intubated	108/558	25	×
Avg	8.0 ± 5.6	–	94 ± 24/681 ± 192	–	–
Gr. 1	5.6 ± 4.7	Intubated	101 ± 25/620 ± 157	–	–
Gr. 2	11.7 ± 5.0	Free-Breathing	82 ± 19/776 ± 214	–	–

Detailed patient, scanning, and imaging information from the 18 subjects included in the study. In the last column, missing values (×) indicate that comparison between SG and ECG triggers was not feasible due to disrupted ECG signals

*ECG* electrocardiogram, *SG* self-gating

Among all 18 ECG traces recorded during the acquisition, 9 (subjects 1, 7, 8, 10–13, 15, 18) had to be excluded from further analysis due to the interferences induced by the free-running GRE acquisition (Table 2). In Additional files 3 and 4, the difference between datasets binned and reconstructed using the SG cardiac signal and using the erroneous ECG triggers can be observed. In the first example from Patient 12 (Additional file 3) where the corrupted ECG signal was used, the motion resolution along the cardiac dimension is insufficient and leads to blurring of myocardial structures. This is in stark contrast to the image reconstructed with the SG signal where sharper tissue borders are observed throughout the cardiac cycle. The second example from Patient 1 (Additional file 4) illustrates an even more compromised situation where cardiac motion could clearly not be resolved in the ECG-gated image, while contraction and relaxation of the same heart is well visualized using SG information. Considering then the 9 remaining datasets (subjects 2–6, 9, 14, 16, 17) with ECG free from artifacts, standard deviation of the difference between corresponding ECG R-peak intervals and SG trigger intervals was found to be 30.7 ± 23.9 ms (Table 2). Finally, among those same 9 subjects, missed ECG triggers were found in 5 ECG traces, ranging from 0.4% (Subject 5) to 11.5% (Subject 9) of the total amount of cardiac cycles during one scan. Conversely, in none of the SG signals from all 18 subjects missed triggers were found.

**Fig. 1:**
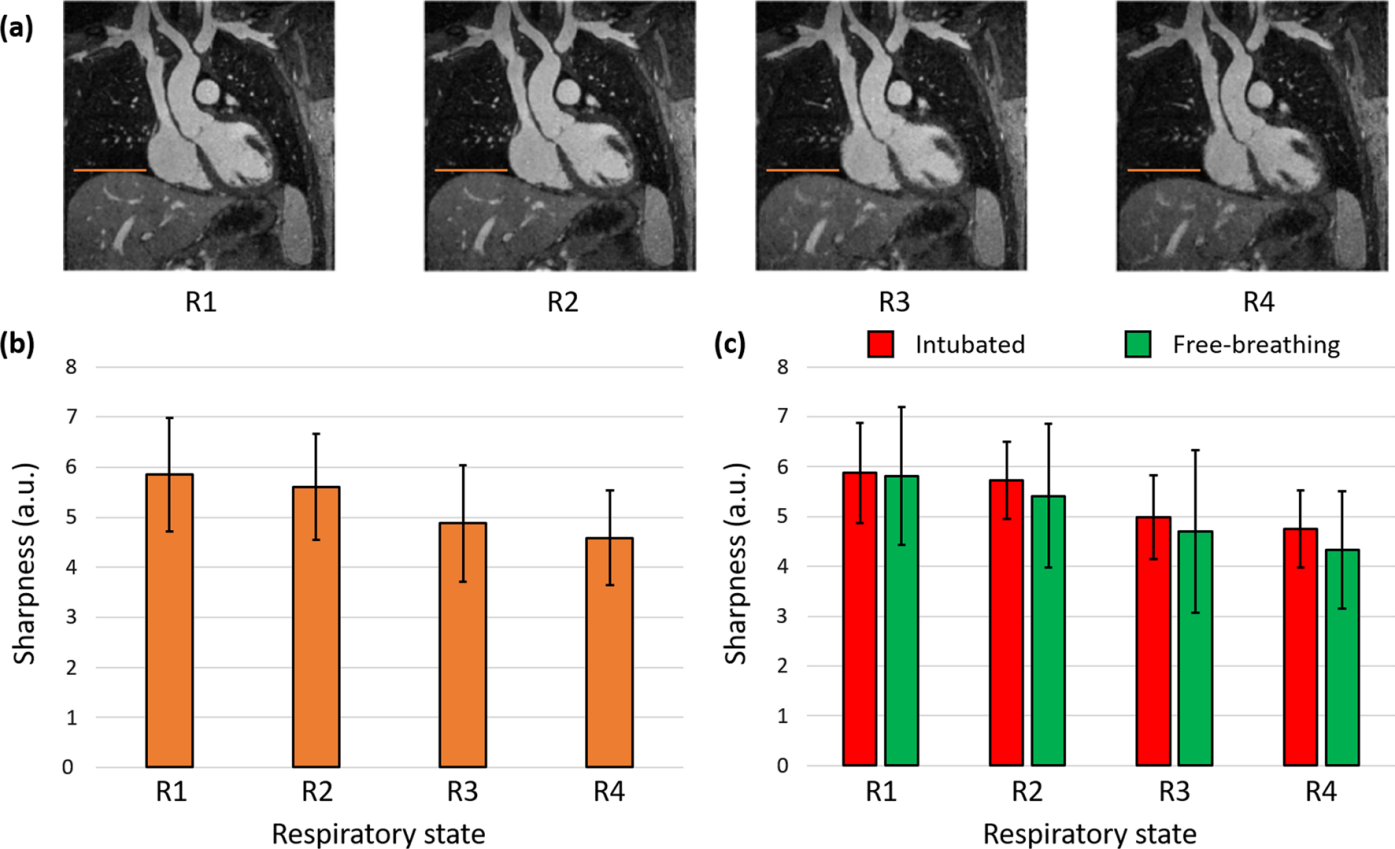
Respiratory motion resolution. **a** Four different respiratory states from a 5D motion-resolved reconstructed dataset are displayed in a coronal view, from end-expiration (R1) to end-inspiration (R4). **b** Average sharpness (over the 18 subjects) of the lung-liver interface for the 4 respiratory states. The difference between R1 and R2 did not reach the statistical significance threshold, and neither did the one between R3 and R4. **c** The interface sharpness values from the plot in **b** are displayed separately for intubated and free-breathing patients. No statistically significant difference was found between the two groups at any respiratory state

Among the resolved respiratory dimension of the 18 reconstructed datasets, sharpness of the lung-liver interface systematically decreased from the end-expiration bin R1 (sigmoid slope $\theta$_*R1*_ = 5.9 ± 1.1) to that at end-inspiration (bin R4, $\theta$_*R4*_ = 4.6 ± 0.9, p < 10^–4^), with images in R1 showing the sharpest interface in 15/18 cases, and those in R4 the most blurred interface in 12/18 cases. More detailed results are presented in Fig. 1. When comparing sharpness between *Group 1* (patients with respiratory intubation, subjects: 1, 3–6, 10, 11, 14, 16–18) and *Group 2* (free-breathing patients, subjects: 2, 7–9, 12, 13, 15), no significant difference was found at any of the 4 resolved respiratory levels (p_R1_ = 0.93, p_R2_ = 0.61, p_R3_ = 0.67, p_R4_ = 0.42) (Fig. 1c).

**Fig. 2:**
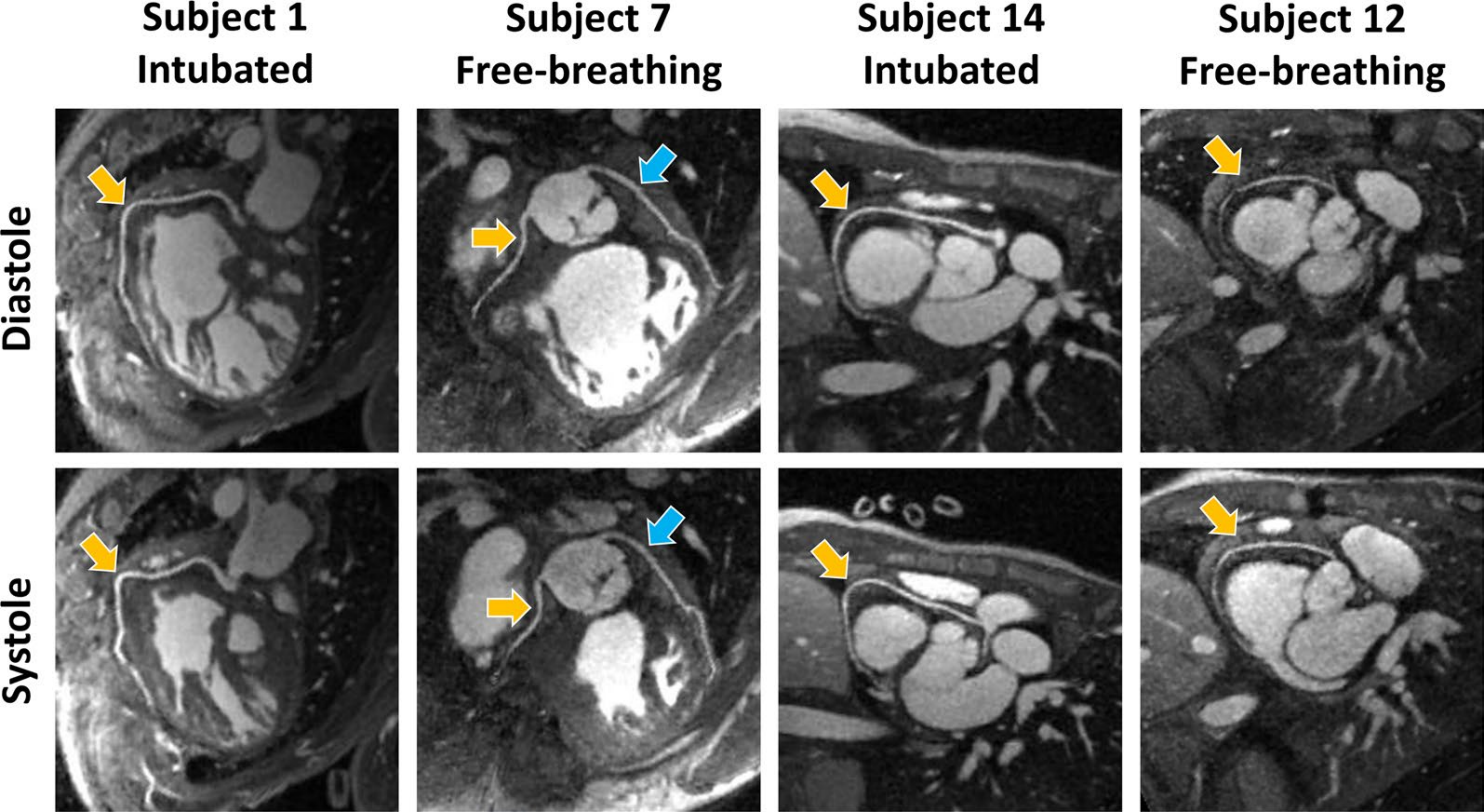
Coronary reformats. Diastolic and systolic coronary reformats for 2 intubated and 2 free-breathing subjects. Yellow arrows indicate the right coronary artery (RCA), while light blue arrows indicate the left main (LM) and left anterior descending coronary artery (LAD; LM + LAD). From a qualitatively point of view, it can be observed how image quality and coronary vessel conspicuity appear similar in the two cardiac phases. Additionally, image quality is comparable between intubated and free-breathing patients

**Fig. 3:**
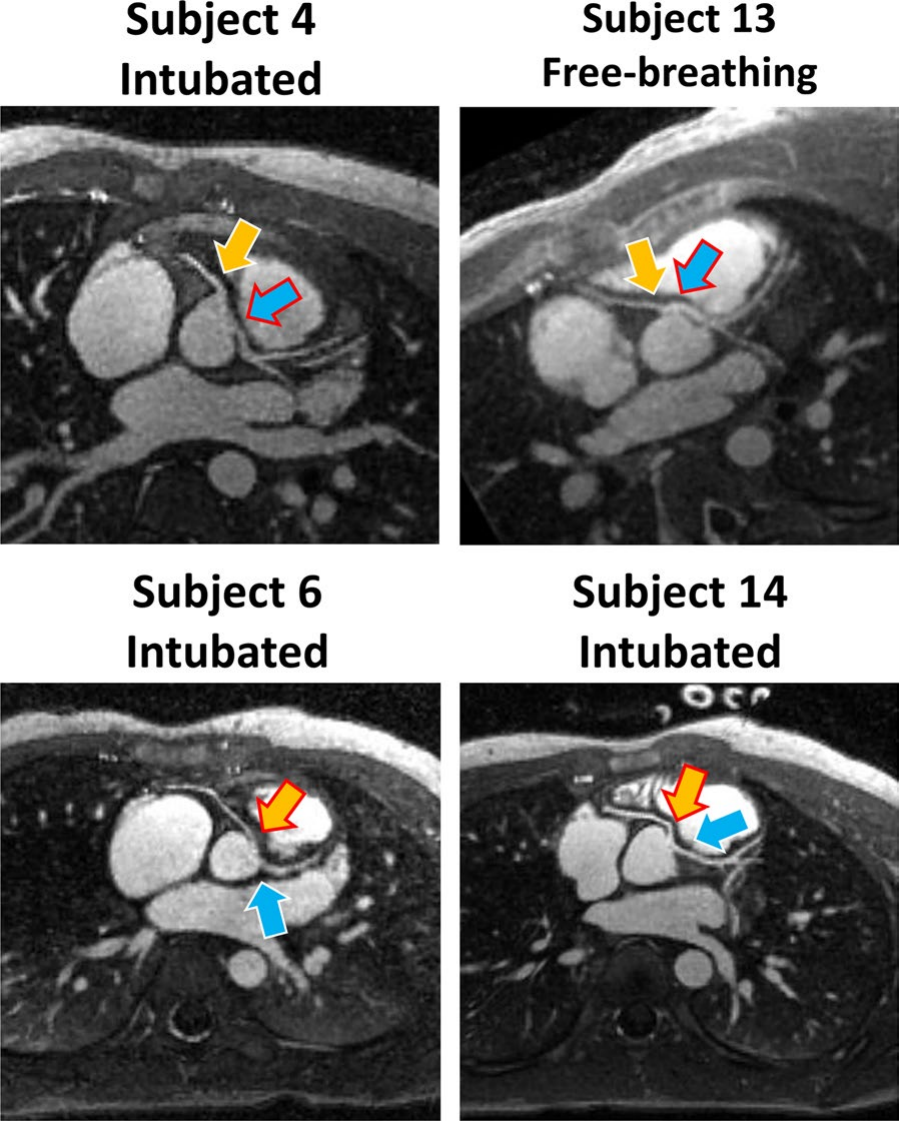
Anomalous coronary anatomy. Coronary reformats for 4 different subjects for simultaneous visualization of RCA ostium (yellow arrow) and LM artery ostium (light blue arrow). The arrows with red outline highlight anomalous coronary vessel anatomy. In particular, subjects 4 and 13 show anomalous LM coronaries originating from the right sinus, while subjects 6 and 14 present anomalous RCA coming out of the left coronary sinus. Overall, the anomalous vessel anatomy is well demonstrated in both intubated and free-breathing patients

Examples of reformatted coronary arteries extracted from end-expiratory systolic and diastolic bins from three different subjects are shown in Fig. 2. Similarly, examples of anomalous coronary artery ostia from four different patients are displayed in Fig. 3, with cases of LM originating from the right sinus and the RCA originating from the left sinus. Among the 4 coronaries considered (RCA, LM, LAD, and LCx) from the 18 subjects, both in systole and diastole, hence resulting in 144 total vessel images analyzed, the origin of the coronary was visible in 130/144 cases (90%), with 82/88 (93%) in intubated patients and 48/56 (86%) in free-breathing patients. Among the visible coronaries, the measured sharpness of the RCA was 45.8 ± 5.4% in systole and 45.2 ± 8.1% in diastole (p = 0.67), the LM + LAD sharpness was 45.1 ± 5.3% in systole and 47.0 ± 10.8% in diastole (p = 0.42), and for the LCx, it was measured 44.5 ± 7.9% in systole and 48.8 ± 7.8% in diastole (p = 0.19). After dividing the measured vessel sharpness into intubated and free-breathing patients, no statistically significant difference was found in the comparison between the two groups (all p > 0.07). Among the same coronaries, the visible length of the RCA was 6.6 ± 2.6 cm in systole and 6.8 ± 2.8 cm in diastole (p = 0.36), the LM + LAD was 5.5 ± 2.5 cm in systole and 6.1 ± 3.1 cm in diastole (p = 0.33), and the LCx was 3.1 ± 1.9 cm in systole and 2.8 ± 1.8 cm in diastole (p = 0.40). Comparing the measured vessel length between *Group 1* and *Group 2*, no significant difference was found except for the RCA, where the visualized length was significantly higher in intubated patients than in free-breathing patients, both in systole and diastole: *L*_*G1,syst*_ = 7.3 ± 2.8 cm vs *L*_*G2,syst*_ = 5.1 ± 1.1 cm (p = 0.048) and *L*_*G1,diast*_ = 7.7 ± 2.9 cm vs *L*_*G2,diast*_ = 5.0 ± 1.7 cm (p = 0.041). Detailed length and sharpness values are presented in Table 3.

**Table 3:** Coronary artery vessel quantification

	Coronary origin visibility							
	RCA		LM		LAD		LCx	
	Systole	Diastole	Systole	Diastole	Systole	Diastole	Systole	Diastole
All subjects	17/18	14/18	17/18	18/18	17/18	17/18	15/18	15/18
Group 1	11/11	9/11	11/11	11/11	11/11	11/11	9/11	9/11
Group 2	6/7	5/7	6/7	7/7	6/7	6/7	6/7	6/7

	Coronary vessel length (cm)					
	RCA		LM + LAD		LCx	
	Systole	Diastole	Systole	Diastole	Systole	Diastole
All subjects	6.6 ± 2.6	6.8 ± 2.8	5.5 ± 2.5	6.1 ± 3.1	3.1 ± 1.9	2.8 ± 1.8
Group 1	7.3 ± 2.8	7.7 ± 2.9	5.4 ± 2.9	5.3 ± 2.9	2.9 ± 2.2	2.7 ± 2.0
Group 2	5.1 ± 1.1*	5.0 ± 1.7*	5.7 ± 1.9	7.6 ± 3.0	3.3 ± 1.3	2.9 ± 1.6

	Coronary vessel sharpness (%)					
	RCA		LM + LAD		LCx	
	Systole	Diastole	Systole	Diastole	Systole	Diastole
All subjects	45.8 ± 5.4	45.2 ± 8.1	45.1 ± 5.3	47.0 ± 10.8	44.5 ± 7.9	48.8 ± 7.8
Group 1	46.2 ± 3.5	44.5 ± 6.4	46.9 ± 4.5	49.0 ± 11.6	45.5 ± 7.1	48.8 ± 9.5
Group 2	45.1 ± 8.7	46.6 ± 11.6	41.6 ± 5.4	43.3 ± 8.9	42.6 ± 9.7	48.8 ± 4.8

Detailed assessment of coronary vessels from the 18 subjects included in the study are collected. Results for visibility of the coronary ostia, vessels sharpness, and vessel length are listed. Asterisks indicate statistically significant differences in vessel measurements between Group 1 (intubated patients) and Group 2 (free-breathing patients), which appears only in the visible length of RCA

*LAD* left anterior descending coronary artery, *LCX* left circumflex coronary artery, *LM* left main coronary artery, *RCA* right coronary artery

## Discussion

A previously reported fully SG free-running framework for cardiac and respiratory motion-resolved 5D imaging of the whole heart was tailored for—and successfully applied to—a cohort of pediatric CHD patients injected with ferumoxytol contrast agent. Apart from exchanging the bSSFP module with GRE and removing fat suppressions and ramp-up pulses, no further adjustments were made, and the proposed imaging technique provided effective cardiac and respiratory motion-resolution, strong blood signal, and overall good definition of the cardiac anatomy with high contrast between the blood-pool and the myocardium. It is noteworthy that a high detail visibility of anatomical structures such as the coronary arteries was obtained in a cohort of CHD pediatric patients, where CMR imaging is often more challenging, and where small structures, high heart rates, and complex anatomy often occur. Moreover, the proposed framework proved to work in a simple “push-button” manner even for a wide range of body sizes, heart rates, clinical questions, and both with or without anesthesia and intubation.

Successful attempts to provide an efficient, comprehensive, and easy-to-use approach to ferumoxytol-enhanced motion-resolved whole-heart imaging in pediatric patients have been reported by Han et al. with the 4D MUSIC technique and its further development to 4D ROCK MUSIC [3, 5, 7]. In this present work, we aimed at adapting and testing a previously developed free-running framework for ferumoxytol contrast-enhanced cardiac pediatric imaging, exploiting the favorable properties of efficiency, flexibility, and simplicity of the free-running approach. At the same time, we wanted to preserve some of the important characteristics of 4D ROCK MUSIC including the high spatial resolution, a large FOV coverage, and relatively short acquisition times. In particular, both techniques provide 3D isotropic submillimeter spatial resolution (between 0.8^3^ and 1^3^ mm^3^ for 4D ROCK MUSIC vs. 0.9^3^ to 1.1^3^ mm^3^ for 5D free-running), they allow the FOV volume to encompass the whole heart (4D ROCK MUSIC: 480 × 280 × 140 mm^3^ vs. 5D free-running: 192 × 192 × 192 mm^3^ when at 1^3^ mm^3^ resolution), and they operate at similar acquisition durations (4–6 min for the 4D ROCK MUSIC vs. 7–8 min for 5D free-running).

Despite these similarities, the temporal resolution obtained with the free-running framework has been improved. While the previously published ROCK-MUSIC approach included 9 cardiac phases that are defined prior to the data acquisition, resulting in an average temporal resolution of over 60 ms in a cohort of pediatric patients with a 106 ± 12 bpm heart rate [7], the proposed free-running approach permits a fully flexible and retrospective selection of the number of cardiac phases that are reconstructed, which led to 13–24 3D cine frames with a window width as short as 25 ms for patients with heart rates above 100 bpm. The opportunity to select the amount of reconstructed cardiac phases retrospectively and flexibly in a patient specific manner may represent a distinct improvement for comprehensive and personalized cardiac motion-resolved imaging. In principle, retrospective selection of temporal resolution could also be applied using other techniques such as ROCK-MUSIC. Considering the extended period during which ferumoxytol remains in the blood pool, a side-by-side comparison between free-running 5D CMRA and ROCK-MUSIC [7] would be feasible, timely, and quite instructive at this juncture. Additionally, a comparison of these techniques which employ radial and Cartesian sampling respectively would be useful in determining the relative effects of eddy currents and off-resonance artifacts in these two sampling schemes. For both ROCK-MUSIC and free-running 5D imaging, the maximum achievable temporal resolution is limited by the repetition time, flexibility of the k-space sampling scheme (i.e., binning of individual lines versus interleaves), and the underlying signal-to-noise. Similarly, for the respiratory motion-resolved component of 5D imaging, the number of reconstructed respiratory phases can be retrospectively chosen to minimize blur provided enough signal is present within the total number of acquired lines. In the present study, and based on previous work, 4 respiratory bins were sufficient to ensure a stable end-expiratory phase. However, while not explored here, 5D imaging enables the assessment of the cardiac anatomy and function in different respiratory stages. Under these circumstances it may be that more respiratory bins are needed to resolve the more variable end-inspiratory phase or require inclusion of intra-bin motion correction [23]. Overall, there is an inherent trade-off between total scan and reconstruction time and the achievable cardiac and respiratory temporal resolution. While we demonstrate that the combination of compressed sensing and ferumoxytol enables a two-fold improvement in temporal resolution (Additional file 1) relative to previous studies [8, 17], future works should systematically investigate what the boundaries of temporal resolution for 5D imaging are, as well as define new strategies to find the optimal number of bins to balance and minimize undersampling artifact on the one hand and motion blurring on the other. Concurrently, the CS reconstruction would also benefit from further optimization of the regularization parameters, which in this study were manually transposed from previous experiences in 5D imaging of healthy adult subjects without contrast agent.

The radial trajectory used in this study leads to an isotropic FOV. Unlike Cartesian sampling, the individual readout, phase-encode, and slice-encode dimensions cannot readily be tailored to the patient but given the two-fold oversampling of radial readouts in all directions, wrap-around artifacts are far less of a concern compared to Cartesian and therefore the field-of-view does not need to be adjusted for each patient, potentially reducing scan planning, and improving overall efficiency.

The free-running framework includes the reconstruction of the fifth dimension (in addition to x–y–z-cardiac dimensions), resolving 4 different motion states of the respiratory cycle for each cardiac phase. While this provides us with the opportunity to visualize 3D cine images at different respiratory levels, this also affords another advantage: instead of discarding data that were acquired outside of a small gating window at end-expiration (leading to a respiratory gating efficiency of about 40% for the 4D ROCK MUSIC approach), all the acquired data contribute information to the final image reconstruction of free-running datasets, increasing the efficiency to 100%. This also implies that scanning time doesn’t need to be manually and prospectively adjusted to the specific patient. Further, unpredictable respiratory patterns can be flexibly accommodated by the retrospective approach to motion resolution. In fact, neither respiratory patterns nor heart rate have a direct influence on scanning time that always remains constant for our proposed approach. This means that this protocol may more easily be integrated into a clinical routine exam. Finally, the reconstruction of different respiratory motion states provides a yet to be exploited opportunity for co-registration using motion fields [24]. This could potentially be aimed at further SNR enhancement, improved spatial resolution, or acquisition acceleration.

The use of ferumoxytol as a CMR contrast agent provides a significant T1-shortening effect in the blood pool that may particularly benefit GRE sequences, which enables imaging with high spatial and high temporal resolution as demonstrated by the current study. Still, a number of limitations must also be considered. Slow infusion of ferumoxytol is necessary to avoid potential anaphylactic reactions which in turn prolongs patient preparation. Additionally, unlike gadolinium, ferumoxytol remains primarily intravascular and may therefore not be easily used for perfusion and delayed enhancement imaging. Considering these limitations, the free-running framework may offer additional advantages in that it provides a comprehensive evaluation of the structure and anatomy of the heart in a predictable scan time creating a potential trade-off between preparation time and actual time in the scanner. Furthermore, while the current study used ferumoxytol, previous studies have demonstrated free-running CCMRA with native contrast and therefore, the current results with ferumoxytol motivate further study of free-running CCMRA in a pediatric population without the use of contrast agents. Conversely, in the current study, strong flow dephasing artifacts were present as demonstrated by Additional file 1. To reduce these artifacts, an ultrashort echo time GRE approach may be useful, but would likely require the continued use of contrast agents due to the poor blood to myocardium contrast obtained with native GRE. On the other hand, and with the flexible a posteriori selection of the time point in the cardiac cycle, images with little or no flow artifacts may freely be selected.

In the current study, half of all subjects had significantly corrupted ECG traces as confirmed by both visual inspection of the raw signals as well as the impact on subsequent reconstructions. This result underscores the utility of the cardiac SG approach which worked well in all patients. At higher field strengths, an alternative to ECG may become even more important. Still, an additional hardware-based gating device that is unaffected by gradient switching such as pulse oximetry or pilot tone [25] may also be useful to automate the process of determining artifactual ECG or SG triggers.

To our knowledge, an uninterrupted, motion-resolved, ferumoxytol-enhanced free-breathing CCMRA without tracheal intubation, anesthesia and controlled ventilation has not been reported in a pediatric CHD cohort. While comparing the free-breathing group to their intubated counterpart, most of the quantitative measurements performed on the reconstructed 5D images did not show significant differences, neither in terms of respiratory motion-resolution at the lung-liver interface, nor for the computed length and sharpness of coronary arteries. The only exceptions were represented by the higher percentage of visible coronary ostia (93% in Group 1 vs 86% in Group 2) and the longer RCA measured in patients scanned under controlled ventilation conditions. Even if these results could be confounded by a relatively small number of patients, the large range of age and spectrum of CHD present in the examined cohort, together with a potential age bias in terms of patient selection for free-breathing scanning (younger patients tend to be less cooperative, hence requiring anesthesia more often), these results may still suggest that the more regular and controlled respiratory pattern of intubated patients may be advantageous in younger pediatric subjects. Additionally, all patients were visually monitored throughout the scan and no instances of significant bulk patient motion were observed in the free-breathing cohort. This was further confirmed by inspecting the SI projections which showed periodic cardiac and respiratory motion without the expected jumps or drifts in signal intensity that typically accompany bulk movement. As a result, we observed high quality images in even our very young free-breathing patients (Additional file 5). In a larger cohort of free-breathing patients where bulk movement may be a concern, a more sophisticated navigator strategy may be needed to compensate for bulk movement. Nevertheless, we believe that the 5D CCMRA approach presented here offers a distinct opportunity for free-breathing data acquisition without intubation in selected pediatric patients that significantly simplifies patient setup, improves ease-of-use, and that may contribute to improved patient comfort in a pediatric CHD cohort in general. The overall quality of all free-breathing subjects is demonstrated by Additional file 6. At this point, we believe future research should further compare imaging performances in free-breathing and intubated patients within a prospective study, where a broader cohort with matching age, gender, and indication is considered. Moreover, after demonstrating the feasibility and utility of the proposed framework in CHD patients with ferumoxytol enhancement, future work should also address the limitations of the current study, by extending the technique to pediatric 5D CCMRA with native contrast using bSSFP [8, 26, 27], by considering gold standard comparisons, and further evaluation of the diagnostic ability.

In summary, in this study we proposed a whole-heart fully self-gated motion-resolved imaging approach that could significantly improve the ease-of-use and extend the dissemination of CCMRA in CHD pediatric patients. The efficiency and flexibility of the free-running acquisition and reconstruction framework, associated to the favorable properties and excellent signal provided by ferumoxytol contrast agent, have the potential to challenge existing paradigms, which usually include lengthy and complex scan planning procedures, synchronization of data acquisition with the ECG, and repeated breath-holds during respiratory intubation and anesthesia. Such an approach may potentially improve patient comfort, while simultaneously reducing the patient set up time, operator dependency, as well as motion artifacts sometimes correlated to disrupted respiratory navigators and ECG triggering. In particular, it has been shown that despite uninterrupted acquisition sequences can at times interfere and degrade the information recorded from ECG devices, the proposed SG strategy can robustly provide physiologic motion information for cardiac resolved reconstructions. Finally, it can be argued that this efficient and practical paradigm is shifting CMR imaging towards a push-button solution where a retrospective interrogation of the data to best answer clinical questions replaces prospective scan planning and pulse sequence parameter adjustments.

## Conclusions

In conclusion, the fully SG free-running framework for motion-resolved 5D imaging of the whole heart has consistently and successfully been employed in pediatric CHD patients injected with ferumoxytol over a wide range of heart rates, body sizes, and clinical questions. It supports high isotropic spatial resolution and high temporal resolution which is critical to account for smaller anatomical structures and increased heart rates as often present in pediatric patient cohorts. Furthermore, respiratory motion suppression is effective regardless of whether free-breathing or intubation together with anesthesia is used. This is corroborated by the overall ability of the technique to visualize the coronary arteries and the results suggest that free-breathing data acquisition without intubation and without anesthesia may be an option in selected patients.

Ease-of-use, large FOV coverage, short and well-defined acquisition times as well as an overall excellent contrast and definition of cardiac structures are among the benefits of this approach, while no gating devices are required. Addressing many concerns of current techniques in clinical practice and providing a push-button imaging approach, this framework may help simplify its use in pediatric CHD patients.

## Supplementary Information

**Additional file 1.** 5D motion-resolved image. From top to bottom, cardiac dimension, respiratory dimension, and fly-through dimension are displayed, respectively. (Patient 14).

**Additional file 2.** 5D image with variable temporal resolution. Transverse, coronal, and sagittal planes from 5D motion-resolved images displayed along the cardiac dimension. Images are reconstructed from the same dataset (Patient 16) with 8 different temporal resolutions, ranging from 15 ms (30 bins) to 150 ms (3 bins).

**Additional file 3.** Electrocardiogram (ECG)-gated vs self-gated (SG) reconstruction. 5D motion-resolved images from the same dataset. Binning and reconstruction were performed first with the electrocardiogram (ECG) triggers (top row) and then with the SG cardiac signal (bottom row).

**Additional file 4.** ECG-gated vs SG reconstruction. 5D motion-resolved images from the same dataset. Binning and reconstruction were performed first with the ECG triggers (top image) and then with the SG cardiac signal (bottom image).

**Additional file 5.** 5D motion-resolved image. Cardiac and respiratory motion is visualized in a two-year-old (patient 15) under free-breathing conditions.

**Additional file 6. **3D fly-through images from all free-breathing subjects. Cardiac anatomy is well demonstrated in all subjects under free-breathing conditions.

## Data Availability

The datasets used and analyzed during the current study are available from the corresponding author on reasonable request.

## Abbreviations

2D: 2-Dimensional
3D: 3-Dimensional
4D: 4-Dimensional
5D: 5-Dimensional
ADMM: Alternating direction method of multipliers
bSSFP: Balanced steady state free precession
CCMRA: Coronary cardiovascular magnetic resonance angiography
CHD: Congenital heart disease
CS: Compressed sensing
ECG: Electrocardiogram
FOV: Field of view
GRE: Gradient recalled echo
LAD: Left anterior descending coronary artery
LCx: Left circumflex coronary artery
LM: Left main coronary artery
MUSIC: MUltiphase Steady-state Imaging with Contrast enhancement
RCA: Right coronary artery
RF: Radiofrequency
ROCK: ROtating Cartesian K-space
SAR: Specific absorption rate
SG: Self-gating
SI: Superior-to-inferior
